# Allium® bulbar urethral stent (BUS) for severe recurrent urethral stricture in a renal transplant recipient: a minimally invasive graft-preserving solution

**DOI:** 10.1016/j.eucr.2026.103394

**Published:** 2026-03-04

**Authors:** Nicola Schiavone, Marco Finati, Anna Ricapito, Oscar Selvaggio, Giuliano Ciavotta, Dario Troise, Silvia Mercuri, Luciana Antonia Cirolla, Ugo Giovanni Falagario, Giuseppe Carrieri, Barbara Infante, Gaetano Valerio Palella

**Affiliations:** a Department of Urology and Renal Transplantation, University of Foggia, Foggia, Italy; b Nephrology, Dialysis and Transplantation Unit, Advanced Research Center on Kidney Aging (A.R.K.A.), Department of Medical and Surgical Sciences, University of Foggia, 71122, Foggia, Italy; c Division of Renal Medicine and Baxter Novum, Department of Clinical Science, Intervention and Technology, Karolinska Instituted, 141 52, Stockholm, Sweden; d Urology Unit, Department of Molecular Medicine and Surgery, Solna, Karolinska Institutet, Stockholm, Sweden

## Abstract

Urethral strictures in kidney transplant recipients seriously jeopardize graft function, causing urinary stasis and infections.

We report a 54-year-old male kidney transplant recipient with recurrent bulbar urethral stricture, multiple failed urethrotomies, and long-term urinary devices dependency. Recurrent UTIs and graft dysfunction prompted Allium® bulbar stent placement. At 12 months, uroflowmetry improved (Qmax from 7.9 to 9 mL/s), post-void residual dropped (100 mL to 30 mL), and IPSS decreased (25 to 9). Graft function improved (creatinine 3 mg/dL). No hospitalizations occurred.

This case suggests Allium® bulbar urethral stent may offer a safe, minimally invasive alternative in high-risk kidney transplant recipients.

## 1 Introduction

Urethral stricture disease often recurs despite visual internal urethrotomy (VIU), with reported recurrence rates of 30–60% within the first year and dwindling success after multiple procedures.^1^ In renal transplant recipients, even mild obstruction can lead to urine retention, hydronephrosis, recurrent urinary tract infections, and ultimately threaten graft survival. In this delicate population, minimizing invasiveness and preserving unobstructed urinary flow are critical goals. ^2^^,^^3^.

Although urethroplasty is considered the gold standard for the treatment of recurrent urethral strictures, these procedures require adequate surgical expertise and are not without complications. ^4^^,^^5^ Moreover, there are insufficient data on renal transplant recipients — who are chronically immunosuppressed with multiple comorbidities and often have a history of prior surgeries — regarding the outcomes and safety of such reconstructive interventions.

## 2 Device features

**Fig. 1:**
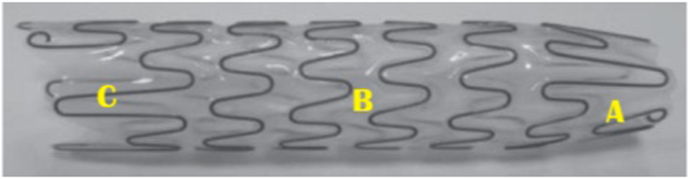
Allium® bulbar urethral stent (BUS). **A**: soft sphenteric segment. **B:** High radial force body. **C**: Soft distal segment.

**Fig. 2:**
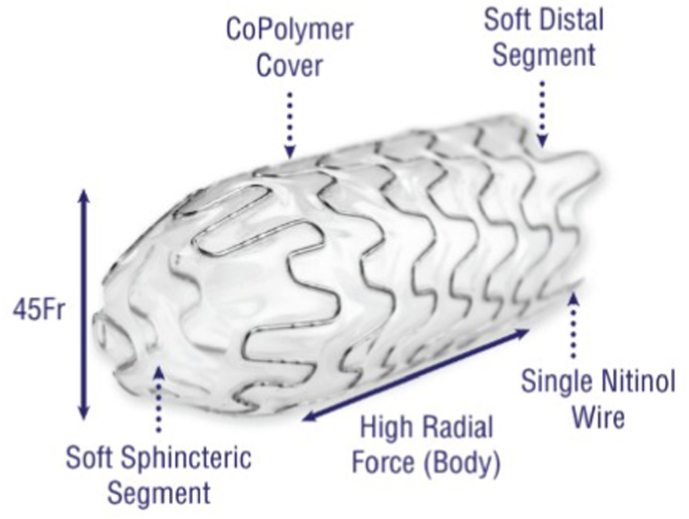
Allium® bulbar urethral stent (BUS) features.

The Allium® Bulbar Urethral Stent (BUS) is a fully covered, self-expandable temporary stent specifically developed for the management of recurrent bulbar urethral strictures. It features a super-elastic nitinol coil with a biocompatible polymer covering that prevents tissue ingrowth and reduces encrustation, mucosal hyperplasia, and stone formation. The stent is available in multiple lengths with a large 45 Fr caliber, allowing it to act as a mold to maintain a wide urethral lumen, while a dynamic segment near the sphincter preserves sphincter function and minimizes incontinence. BUS is intended for extended indwelling periods (up to approximately three years) and is designed for easy endoscopic insertion and safe removal, even after prolonged placement, through a unique unraveling feature that facilitates non-traumatic retrieval. Clinical studies have demonstrated its safety, high rates of urethral patency, and effectiveness in improving voiding function when used following urethrotomy and/or scar tissue resection (Fig. 1, Fig. 2).

## 3 Case presentation

A 54-year-old male with a renal transplant in 2012 was referred for recurrent UTIs and declining graft function in the setting of known urethral obstruction. His history included a prior kidney transplant in 2000 (later explanted due to sepsis). Since adolescence he had signs of obstructive uropathy, elevated post-void residuals, cystoscopically documented anterior urethral strictures, and bladder neck sclerosis (Table 1).

**Table 1:** Patient features.

**Age**	54
**Transplant History**	Kidney transplant in 2012, non-functional kidney transplant in 2000, recurrent urethral strictures
**Cistoscopy**	Bulbar stricture
**Devices carrying pre-operatively**	Nephrostomy, sovrapubic catheter
**Treatment**	Allium® bulbar urethral (BUS) stenting
**Follow up**	Improved IPSS, RPM and Qmax at 12 months

After transplantation, he developed recurrent UTIs early and progressively worsening renal function (creatinine up to 4 mg/dL), exacerbated by cardiovascular comorbidities. Over ten years, multiple internal urethrotomies were attempted but failed due to immediate re-stenosis. Poor adherence to urologic device management led to numerous repositioning surgeries of nephrostomies and suprapubic catheters. At presentation he depended on a suprapubic catheter.

**Fig. 3:**
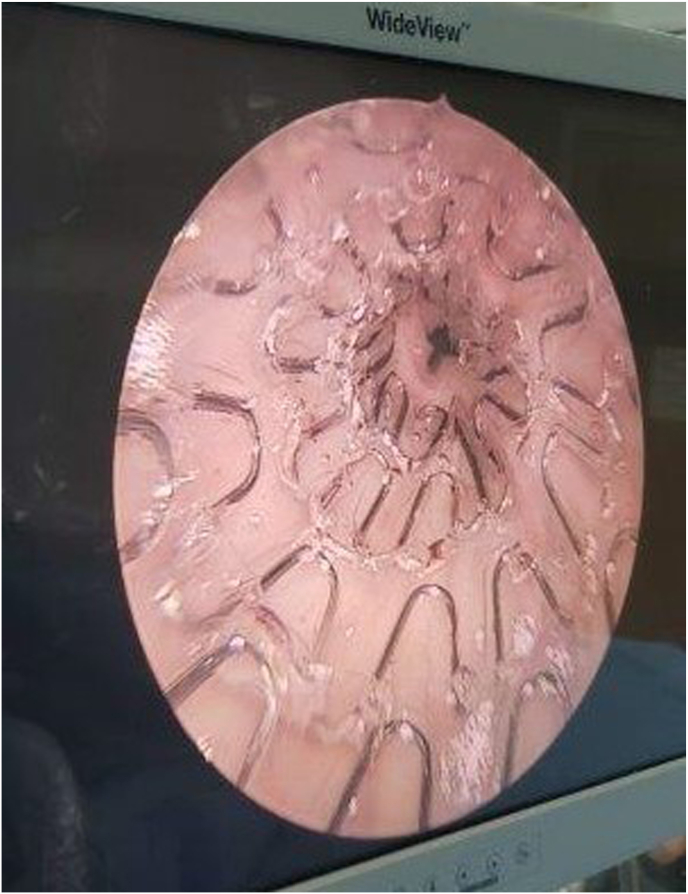
Allium® bulbar urethral stent (BUS) placed correctly.

Given repeated failures and procedural risk, in February 2025 a self-expandable Allium® bulbar urethral stent was placed under endoscopic guidance (Fig. 3). The procedure was uneventful. Follow-up was at 12 months postoperatively using uroflowmetry and the International Prostatic Symptoms Score (IPSS) questionnaire. Compared with the first postoperative day baseline, Qmax improved from 7.9 to 9 mL/s, post-void residual decreased from 100 mL to 30 mL, and IPSS fell from 25 to 17. During the 12-month follow-up, the patient had no fever, no UTIs, and no hospital admissions. At 12 months after the surgery, the Qmax remained stable, the PVR was almost completely absent (25 ml out of 250 ml voided volume), and the IPSS score further decreased to 9. Serum creatinine remained at 3 mg/dL.

## 4 Literature review

Evidence of using self-expander urethral stent in kidney transplant recipients remains silent.

No studies report the use of Allium® bulbar urethral stents in renal transplant patients with urethral strictures. A 2024 retrospective study of 497 kidney transplant recipients describes standard surgical treatments and 12-month outcomes, but does not explore non-conventional alternatives like Allium™ bulbar stents when traditional methods fail.^6^

## 5 Discussion

Managing urethral stricture in renal transplant patients requires balancing efficacy with graft safety and procedural risk. Conventional approaches, including repeated VIU or urethroplasty, often carry significant morbidity and may jeopardize graft function or provoke infection in immunosuppressed individuals.

While earlier generation permanent urethral stents such as the Urolume were associated with significant long-term complications, including epithelial ingrowth and difficult removal, the Allium® bulbar urethral stent is a fully covered, self-expandable device designed for temporary placement. Its polymeric covering prevents tissue ingrowth and allows atraumatic removal, potentially reducing the risk of the severe complications historically reported with permanent stents.

Allium® self-expandable bulbar urethral stents offer continuous luminal support while minimizing tissue ingrowth and bacterial colonization. Use of these devices is emerging for recurrent urethral strictures with promising results^7^; however, there are no reports in the literature about their use in kidney transplant patients. In our patient, stent placement restored spontaneous voiding, eliminated the need for a chronic drainage catheter, and improved both objective and subjective voiding parameters—all without compromising graft function.

Long-term dependence on indwelling catheters or nephrostomies in transplant recipients is problematic: these devices predispose to chronic bacteriuria, recurrent UTIs, local trauma, and reduced quality of life. Each infection episode represents a risk not only for patient morbidity but also for graft impairment and interruption of immunosuppressive therapy. In our patient, catheter dependence had already driven repeated infections and procedural burden; the stent allowed removal of the catheter and interruption of that vicious cycle.

Furthermore, these devices are characterized by relatively straightforward postoperative management and their implantation does not require a steep learning curve, which may facilitate broader adoption even in centers with limited experience in complex urethral reconstruction. This increased accessibility could expand treatment options for transplant recipients with urethral strictures, particularly in settings where specialized expertise in urethral pathology is less readily available, without imposing a significant additional procedural burden.

Economic considerations also support limiting repetitive interventions and hospitalizations. Chronic device maintenance, infection treatment, and repeated surgeries consume healthcare resources. While a Allium® bulbar urethral stent has an upfront cost, it may be cost-effective if it reduces hospital admissions, device exchanges, and infection-related procedures over time.

In this case, despite a modest improvement in Qmax the stent achieved symptomatic improvement and stabilization of renal parameters over a short period—without complications such as migration, encrustation, or infection. As a minimally invasive surgical therapy, the primary goal was to ensure urethral patency and clinical stability while avoiding major reconstructive surgery rather than achieving maximal flow rates. Although longer-term follow-up and larger series are needed, this outcome suggests that urethral stenting may become a viable salvage option in high-risk transplant recipients where conventional therapies have failed.

## 6 Conclusion

This case underscores the potential of self-expandable urethral stents as a minimally invasive, graft-preserving strategy for complex, recurrent urethral strictures in renal transplant patients. In those with poor compliance, recurrent infection risk, and limited surgical options, stenting may afford stable urinary drainage, symptomatic relief, and protection of graft function—all while reducing procedural burden. Further studies with more patients and long-term follow up are required to validate long-term durability and cost-effectiveness, but early evidence is promising.

## CRediT authorship contribution statement

**Nicola Schiavone:** Writing – original draft, Data curation. **Marco Finati:** Writing – review & editing, Data curation. **Anna Ricapito:** Writing – review & editing, Data curation. **Oscar Selvaggio:** Data curation. **Giuliano Ciavotta:** Data curation. **Dario Troise:** Data curation. **Silvia Mercuri:** Data curation. **Luciana Antonia Cirolla:** Data curation. **Ugo Giovanni Falagario:** Supervision. **Giuseppe Carrieri:** Supervision. **Barbara Infante:** Supervision. **Gaetano Valerio Palella:** Supervision.

## Informed consent statement

Informed consent was obtained from the subject involved in this case report.

## Data availability statement

This data presented in this study are available on request from the corresponding author. The data are not publicly available.

## Funding

This research received no external funding

## Conflicts of interest

All the authors have no conflicts of interest to report.
